# Exome sequencing findings in children with annular pancreas

**DOI:** 10.1002/mgg3.2233

**Published:** 2023-08-28

**Authors:** Georgia Pitsava, Nathan Pankratz, John Lane, Wei Yang, Shannon Rigler, Gary M. Shaw, James L. Mills

**Affiliations:** ^1^ Division of Intramural Research, Division of Population Health Research, Eunice Kennedy Shriver National Institute of Child Health and Human Development National Institutes of Health Bethesda Maryland USA; ^2^ Department of Laboratory Medicine and Pathology University of Minnesota Medical School Minneapolis Minnesota USA; ^3^ Department of Pediatrics Stanford University School of Medicine Stanford California USA; ^4^ Department of Neonatology Naval Medical Center Portsmouth Portsmouth Virginia USA

**Keywords:** annular pancreas, cell migration, IQGAP1, malformation, NRCAM

## Abstract

**Background:**

Annular pancreas (AP) is a congenital defect of unknown cause in which the pancreas encircles the duodenum. Theories include abnormal migration and rotation of the ventral bud, persistence of ectopic pancreatic tissue, and inappropriate fusion of the ventral and dorsal buds before rotation. The few reported familial cases suggest a genetic contribution.

**Methods:**

We conducted exome sequencing in 115 affected infants from the California birth defects registry.

**Results:**

Seven cases had a single heterozygous missense variant in *IQGAP1*, five of them with CADD scores >20; seven other infants had a single heterozygous missense variant in *NRCAM*, five of them with CADD scores >20. We also looked at genes previously associated with AP and found two rare heterozygous missense variants, one each in *PDX1* and *FOXF1*.

**Conclusion:**

*IQGAP1* and *NRCAM* are crucial in cell polarization and migration. Mutations result in decreased motility which could possibly cause the ventral bud to not migrate normally. To our knowledge, this is the first study reporting a possible association for *IQGAP1* and *NRCAM* with AP. Our findings of rare genetic variants involved in cell migration in 15% of our population raise the possibility that AP may be related to abnormal cell migration.

## Graphical abstract

Annular pancreas (AP) is a congenital defect of unknown cause in which the pancreas encircles the duodenum. Several theories currently exist. We conducted exome sequencing in 115 affected infants and identified missense variants in two genes IQGAP1 and NRCAM. To our knowledge, this is the first study reporting a possible association of those two genes with AP. Our findings of rare genetic variants involved in cell migration in 15% of our population raises the possibility that AP may be related to abnormal cell migration.

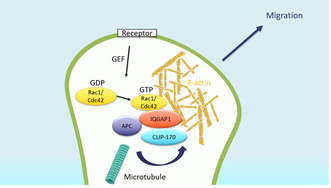

## 1 INTRODUCTION

Annular pancreas (AP; OMIM #167750) is a congenital anomaly in which the descending part of the duodenum is encircled by a ring of pancreatic tissue. It is thought to be due to failure of the ventral pancreatic bud to rotate normally with the duodenum (Nagpal et al., 2019). The exact prevalence cannot be determined as many individuals remain asymptomatic, but older studies have estimated it to be approximately 1 in 10,000 from autopsies and surgical cases (Ravitch & Woods, 1950).

### 1.1 Embryologic development

The pancreas develops from two endodermal buds, the ventral and the dorsal. They develop by the fourth week post‐conception. Around the 7th week, the ventral bud rotates clockwise, fuses with the dorsal bud and forms the adult pancreas (Patra et al., 2011).

### 1.2 Current theories

It has been generally accepted that the origin of AP lies in the ventral pancreatic bud, but the exact mechanism has yet to be determined. Three major theories have been proposed: (1) Lecco's theory in which the ventral bud adheres to the duodenal wall before rotation (Gross & Tague, 1944; Lecco, 1910; Lehman, 1942; Suda, 1990), (2) Baldwin's theory in which there is persistence and enlargement of the ventral bud (Baldwin, 1910; Moore, 2003; Nobukawa et al., 2000), and (3) Tieken's theory in which hypertrophy of the ventral and dorsal buds form an annular structure (Hill, 1993; Tieken, 1901). So far, none of the proposed theories have provided an adequate explanation of the variable ductal anatomy and no consensus has been reached (Etienne et al., 2012; Fu et al., 2005; Godil & McCracken, 1997; Nobukawa et al., 2000). There may be more than one mechanism for the development of AP as the existing embryological hypotheses cannot adequately explain it.

### 1.3 Genetic implications

Some reports suggest that genetic factors play a role in the development of AP. There are case reports of an affected mother and children (Hendricks & Sybert, 1991; Jackson & Apostolides, 1978; MacFadyen & Young, 1987; Rogers et al., 1993). Affected siblings have been reported as well (Claviez et al., 1995; Lainakis et al., 2005; Montgomery et al., 1971). In addition, AP has also been associated with syndromes such as Down syndrome (Stoll et al., 2015; Torfs & Christianson, 1998), Jacobsen syndrome (Fernandez Gonzalez et al., 2002), VACTERL (Reutter et al., 2014), 1p36 deletion syndrome (Minami et al., 2005), Mitchell–Riley syndrome (Zegre Amorim et al., 2015), and 22q11.2 deletion (Spineli‐Silva et al., 2018). In our study, we performed exome sequencing in newborns with AP to search for genetic factors and to try to better understand its etiology.

## 2 MATERIALS AND METHODS

### 2.1 Study design

The data (including information on birth defects and medical and demographic data) were obtained from the California Birth Defects Monitoring Program (CBDMP), a population‐based surveillance system for collecting information on children born between 1984 and 2014 with congenital malformations (Croen et al., 1991). The study excluded all aneuploid and known syndromic cases of AP. Demographic and diagnostic information from medical records for liveborn and stillborn fetuses (defined as ≥20 weeks gestation) were collected by trained data collectors. Overall, ascertainment for major malformations has been estimated as 97% complete (Schulman & Hahn, 1993). The DNA for the study was obtained from newborn screening filter paper, so only live born infants were included.

Data were de‐identified and personally identifiable information was not provided to the investigators to ensure confidentiality and patient privacy. Demographic characteristics of all births in the same region and time from which the AP infants were identified were obtained from birth certificates. These data included maternal race–ethnicity, maternal age, gestational age at delivery, infant's sex, and birthweight. Differences in demographic factors were assessed by chi‐squared and Fisher's exact tests and associations were estimated by odds ratios (ORs). The protocol for this study, including the state‐approved guidelines for the use of newborn bloodspots, was approved by the California Department of Public Health Institutional Review Board (IRB) 14‐08‐1693 and by the NIH Office of Human Subjects Research Protections. The guidelines permit research use of archived newborn bloodspots without further individual consent.

### 2.2 Specimen processing and sequencing

Residual filter paper after newborn screening was obtained and four 3 mm dried blood spot (DBS) punches were washed at 4C for 18–24 h in PBS with 0.1% BSA. DNA was extracted from each dried DBS punch individually using GenSolve reagents (GenTegra) and purified using QIAmp DNA Blood Mini reagents (Qiagen). After extraction and purification, DNA from the four punches was combined and dried to a final volume of 100 μL prior to quantification by PicoGreen (Molecular Probes). Samples were then further dried to 2 μL.

Exome sequencing of the DNA samples from 115 patients was then performed at the University of Minnesota Genomics Center (UMGC). Targeted capture libraries were generated using the Agilent SureSelect Human All Exon v7 kit and were sequenced on an Illumina NovaSeq SP producing 151 bp paired end reads.

### 2.3 Alignment and genotype calling

FASTQ files were processed using a Genome Analysis Toolkit (GATK) v3.7 based pipeline, including using BWA‐MEM v0.7.17 for alternate contig aware alignment to the hg38 reference genome (GRCh38_full_analysis_set_plus_decoy_hla.fa); Picard Tools v2.6.0 to mark duplicates (Picard, retrieved from http://broadinstitute.github.io/picard/); and GATK for indel realignment, base quality recalibration, genotyping (HaplotypeCaller), variant quality score recalibration, and to split multiallelic sites (Li & Durbin, 2010; McKenna et al., 2010).

### 2.4 Annotation

All variants were annotated for functional impact (putative amino acid changes and predicted deleteriousness, e.g., the combined annotation dependent depletion [CADD] score; Rentzsch et al., 2019) using SnpEff v4.3r (Cingolani et al., 2012), the Ensembl Variant Effect Predictor (McLaren et al., 2016), and ANNOVAR v2018Apr16 (Wang et al., 2010). ANNOVAR was also used to annotate the presence and allele frequency (AF; including ancestry‐specific frequencies) of each variant in several public databases, dbSNP (Sherry et al., 2001) version 151, 1000 Genomes (Genomes Project Consortium et al., 2015), NHLBI GO Exome Sequencing Project 6500 exomes (NHLBI‐ESP, n.d.), Exome Aggregation Consortium, and Genome Aggregation Database (gnomAD; Karczewski et al., 2019).

### 2.5 Specimen quality control

Reported sample sex and relatedness was verified using Peddy v0.4.2 (Pedersen & Quinlan, 2017). Sample quality was assessed with FastQC v0.11.2 (FastQC, retrieved from https://www.bioinformatics.babraham.ac.uk/projects/fastqc/), VerifyBamID's freemix score, and an internal QC pipeline (Jun et al., 2012).

### 2.6 Filtering and prioritization

Our genotyping pipeline identified 479,669 unique variants. Of those 372,783 (77.7%) were deemed high quality (not in a GATK tranche, depth > 10, genotype quality [GQ] >50). Of those, 75,949 affected an amino acid (missense or nonsense variants) and were deemed putatively functional. We then focused on genes in which loss‐of‐function variants were found in more than one individual or in which missense or/and loss‐of‐function variants were found in more than five individuals. Using the gene damage index (GDI; Itan et al., 2015) we prioritized novel and known gene candidates, focusing on genes with mutational burdens in the lowest 75th percentile. Of the 75,949 variants, 10,462 met our primary criteria (maximum allele frequency [AF] ≤0.005 and in a gene with a GDI <75). In addition, all putatively functional (missense or loss‐of‐function) rare variants (AF ≤0.005) in genes previously associated with AP were manually reviewed using the Integrative Genomics Viewer (IGV) v2.4.13 (Robinson et al., 2011), regardless of the quality. Filtering criteria are summarized in Table S1 which specifically describes each of the filtering thresholds applied to nominate the variants and genes of interest.

### 2.7 Variant classification

Variants that met our primary filtering criteria were then evaluated using Franklin Genoox (https://franklin.genoox.com/), an AI‐based interpretation engine that provides classification for single nucleotide variants using the American College of Medical Genetics and Genomics (ACMG) guidelines. According to the ACMG, variants are classified into: class 1 (benign), class 2 (likely benign), class 3 (variants of uncertain significance—VUS), class 4 (likely pathogenic), and class 5 (pathogenic; Richards et al., 2015). Additionally, variants were searched in online public databases, including ClinVar (https://www.ncbi.nlm.nih.gov/clinvar/) and gnomAD (https://gnomad.broadinstitute.org/). For novel candidate variants that were not present in these databases, in silico tools including Sorting Intolerant From Tolerant (Sim et al., 2012; range 0_1; predicted pathogenic 0–0.05) and MutationTaster (probability value 0–1 where 1 indicates a high confidence of the prediction; deleterious; Schwarz et al., 2014) were used to further evaluate predicted pathogenicity of the candidate coding variants (Table S1).

## 3 RESULTS

The prevalence of AP in this study population was 1.7 per 100,000. Demographic characteristics of the cases were compared to the same underlying population of births from which the AP cases were ascertained based on a total of 6,783,570 births in the time period and region (Table 1). Among the 115 cases that were sequenced, 64 (55.6%) were females and 70 (61%) were full term. AP‐affected infants were more frequently African American than the source livebirth population (13% vs. 6.5%; OR = 2.20, 95% CI 1.27–3.81, *p* = 0.011). As expected with many major birth defects, many children were delivered prematurely. Therefore, the percentage of preterm births was higher than in the general population and there was a significantly higher percentage of low birth weight infants among cases (34.8% vs. 5.2%). Accompanying birth defects of AP‐affected infants are shown in Table 2.

**TABLE 1:** Demographics of annular pancreas cases and California birth defects monitoring program live birth controls, 1984–2014.

Characteristic	AP cases (*n* = 115; %)	CBDMP live births (*n* = 6,783,570; %)	Overall *p* value^a^
Maternal race/ethnicity			0.0368
White (including Hispanics)	84 (73)	5,437,181 (80.1)	
African Americans^b^	15 (13)	441,474 (6.5)	0.0110
Asian	12 (10.4)	743,052 (11)	
Other	2 (1.7)	90,486 (1.3)	
Missing	2 (1.7)	71,377 (1.1)	
Maternal age at delivery (years)			
13–19	19 (16.5)	746,015 (11)	0.0579
20–24	36 (31.3)	1,654,888 (24.4)	
25–29	30 (26.1)	1,897,181 (28)	
30–34	18 (15.6)	1,557,088 (23)	
35–55	12 (10.4)	926,479 (13.7)	
Missing	0	1919 (<0.1)	
Gestational weeks^c^			
Preterm (<37)	39 (34)	634,683 (9.4)	
Term (37–43)	70 (61)	5,467,150 (80.6)	
Missing	6 (5.2)	681,737 (10.1)	
Infant sex			
Male	49 (42.6)	3,470,111 (51.2)	0.0975
Female	64 (55.6)	3,313,351 (48.8)	
Missing	2 (1.7)	108 (<0.1)	
Infant birth weight (g)^c^			
500–1499	6 (5.2)	69,735 (1)	
1500–2499	40 (34.8)	353,822 (5.2)	
2500–5000	69 (60)	6,337,255 (93.4)	
Missing	0	22,758 (0.3)	

*Note*: Gestational weeks were not included in the analysis.

^a^ Wald chi‐squared test excluding missing.

^b^ Fisher's exact test comparing African Americans versus White.

^c^ The state only provided data in categories to preserve confidentiality.

**TABLE 2:** Additional diagnoses in infants with annular pancreas. California Birth Defects Monitoring Program (CBDMP), 1984–2014.

Case description and classification in CBDMP	Number of infants^a^ (%)
GI defects: Duodenal atresia	66 (57.4)
Malrotation	32 (27.8)
Ladd bands	10 (8.7)
Duodenal stenosis	14 (12.2)
Tracheo‐esophageal fistula	9 (7.8)
Imperforate anus	8 (7)
Rib cage defects: Anomalous number of ribs	6 (5.2)
Heterotaxy/situs ambiguous/situs inversus	8 (7)
Cardiac defects: Ventricular septal defect	21 (18.2)
Atrial septal defect	13 (11.3)
Patent ductus arteriosus	9 (7.8)
Dextrocardia	7 (6)
Common single ventricle	6 (5.2)
Common AV valve	6 (5.2)
Hypoplastic aortic arch	5 (4.3)
Pulmonary valvular stenosis	5 (4.3)

*Note*: For confidentiality reasons, we summarize other accompanying defects as the following. Fewer than five patients were observed to have: microtia, micrognathia, cleft lip/palate, pectus excavatum, anomalous vertebrae in thoracic spine, anomalous upper ribs, aortic valve insufficiency, single semilunar valve, tricuspid valve insufficiency, coarctation of the aorta, AV valve insufficiency, hypoplastic L heart syndrome, mitral valve atresia, common atrium, truncus arteriosus, hypoplastic pulmonary valve, hypoplasia of pulmonary artery, one pulmonary artery, hypoplastic R ventricle, tetralogy of Fallot, pulmonary art stenosis, mitral valve insufficiency, dysplastic AV valve, bicuspid aortic valve, mitral valve stenosis, TAPVR, bilobed R lung, absent main pulmonary trunk, abnormal trachea and bronchi, hypoplastic lungs, hypospadias, undescended testicles, microcephaly, macrocephaly, R large finger, radial clubhand, small trapezoidal middle segment of R hand, partial fusion of lumbosacral junction, equinovarus, finger aplasia, hypoplastic toe distal phalanges, digitalized thumbs, hypoplasia of the ossification center of the middle phalanx of the ring fingers, four fingers on L, no index finger R, triphalangeal thumb, bilateral radial hypoplasia, fusion of radius and ulna bl, underdeveloped sacrum on the R, absent first metacarpal L, R thumb hypoplastic, poorly formed phalangeal bones in L thumb, triphalangeal R thumb, micromelia, polydactyly of small finger of R hand, distally placed thumb, bifid scrotum, short phallus, penile chordee, persistent urachus, didelphic uterus, bicornate uterus, or small R kidney with cysts, L ureterocele, abnormal shaped bladder, absent L kidney, renal dysgenesis, renal cysts, deformed bladder, narrowing of distal urethra, hypoplastic kidneys, asplenia, polysplenia.

Abbreviations: AV, atrioventricular, GI, gastrointestinal.

^a^ Cases can be counted in more than one category.

Of the total of 115 cases sequenced, 91 passed sample quality filters (FREEMIX <0.05). Of the 91, 78 cases harbored at least one variant passing our variant thresholds. Based on our criteria for prioritizing variants (≥2 loss‐of‐function variants or ≥6 loss‐of‐function or missense variants) two genes emerged as candidates for AP, *IQGAP1* (IQ motif‐containing GTPase activating protein 1), and *NRCAM* (neuronal cell adhesion molecule). Seven cases had a single heterozygous missense variant in *IQGAP1* (none were recurrent). All seven variants were present in the gnomAD database and their allele frequencies are noted in Table 3. None of these variants are present in ClinVar. In addition, five of seven variants are predicted by CADD score to be within the top 1% of deleterious variants in the genome. Seven other infants had a single heterozygous missense variant in *NRCAM* (none were recurrent), five of them with CADD scores >20 (Table 3) Only one of the seven variants was present in gnomAD, with allele frequency 0.0003247. Neither of these genes was associated with any previously described syndromes. We also looked at known databases (including OMIM, GeneCards and Uniprot) but they did not provide any additional information related to AP. According to the criteria for variant classification from the ACMG, all variants in both genes are classified as VUS except one that is classified as likely benign and one as likely pathogenic. The classification of each specific variant is described on Table 3.

**TABLE 3:** Variants in IQGAP1 and NRCAM meeting our primary filtering criteria.^a^

Case	Gene	Variant	Locus‐reference allele	[Allele 1, Allele 2]	GQ	AD	AF^b^	CADD	GDI (%)	ACMG classification
1	*IQGAP1*	p.Ser19Phe	15:90390774‐C	[C, T]	99	[5, 15]	0.0013	22.8	44.4	VUS
2	*IQGAP1*	p.Asp353Glu	15:90448718‐T	[T, G]	99	[8, 7]	0.0004	1.8	44.4	VUS
3	*IQGAP1*	p.Thr435Ala	15:90452915‐A	[A, G]	99	[7, 9]	0.00007	16.7	44.4	VUS
4	*IQGAP1*	p.Val525Met	15:90454513‐G	[G, A]	99	[25, 21]	0.003	24.6	44.4	VUS
5	*IQGAP1*	p.Pro1157Ser	15:90482099‐C	[C, T]	99	[39, 44]	0.0002	33.0	44.4	LP
6	*IQGAP1*	p.Met1231Ile	15:90483498‐G	[G, A]	99	[18, 9]	0.0041	21.9	44.4	VUS
7	*IQGAP1*	p.Ala1412Thr	15:90487568‐G	[G, A]	99	[30, 20]	0.0003	23.4	44.4	VUS
33	*NRCAM*	p.Glu1087Lys	7:108168331‐C	[C, T]	99	[4, 6]	0.0018	19.4	47.2	LB
32	*NRCAM*	p.Asp731Ala	7:108184305‐T	[T, G]	99	[20, 25]	0.0004	25.0	47.2	VUS
30	*NRCAM*	p.Thr662Ala	7:108189648‐T	[T, C]	99	[28, 27]	0.0035	22.8	47.2	VUS
31	*NRCAM*	p.Thr640Ala	7:108189714‐T	[T, C]	99	[42, 61]	0.0001	26.4	47.2	VUS
35	*NRCAM*	p.Ala621Thr	7:108191753‐C	[C, T]	99	[25, 16]	0.0002	35.0	47.2	VUS
12	*NRCAM*	p.Thr518Met	7:108194321‐G	[G, A]	99	[13, 4]	0.0001	28.6	47.2	VUS
34	*NRCAM*	p.Val441Ile	7:108197968‐C	[C, T]	99	[14, 20]	0.0001	18.7	47.2	VUS

*Note*: Transcripts used for annotation are provided in Table S1 with Integrative Genomics Viewer screenshots.

Abbreviations: ACMG, American College of Medical Genetics; AD, allelic depth for the [Allele1, Allele2]; AF, allele frequency; CADD, combined annotation dependent depletion score; GDI, gene damage index; GQ, genotype quality; LB, likely benign; LP, likely pathogenic; VUS, variant of unknown significance.

^a^ AF < 0.005, GDI < 75th percentile.

^b^ Maximum AF observed in public databases for any subpopulation.

The likely pathogenic variant (c.3469C > T, p.Pro1157Ser) has a relatively low frequency in gnomAD (0.0002). It notably falls in *IQGAP1*, a gene expressed during human pancreatic development. In addition, this variant disrupts the RAS‐GTP activation domain, which supports its potential damaging effect.

We also looked for variants in genes previously reported to be associated with AP. We checked *RFX6*, *FOXF1*, *PDX1*, *IHH*, and *SHH*, and we identified (≥1 loss‐of‐function or missense variants, AF ≤0.005) two rare heterozygous missense variants in the *PDX1* gene (p.Ala104Asp) and the *FOXF1* gene (p.Met257Ile; Table 4). Next, we checked for X‐linked recessive genes and we identified three variants in *IQSEC2* (p.His1246_His1247del, p.Pro1109Leu, and p.Gly115Cys; Table 5). No compound heterozygotes or autosomal homozygotes passed our IGV review. Additional variants that passed our criteria of the autosomal dominant model are noted on Table S2.

**TABLE 4:** Variants in genes previously reported to be associated with annular pancreas that met filtering criteria^a^, ordered by gene damage index.

Case	Gene	Variant	Locus‐reference allele	[allele 1, allele 2]	GQ	AD	AF^b^	CADD	GDI (%)
8	*FOXF1*	p.Met257Ile	16:86511340‐G	[G, T]	99	[24, 25]	0.0001	12.4	36.9
9	*PDX1*	p.Ala104Asp	13:27920449‐C	[C, A]	99	[28, 32]	0	7.7	55.5

*Note*: The following genes previously reported to be associated with AP were evaluated: *RFX6*, *FOXF1*, *PDX1*, *IHH*, *SHH*.

Abbreviations: AD, allelic depth for the [Allele1, Allele2]; AF, allele frequency; CADD, combined annotation dependent depletion score; GDI, gene damage index; GQ, genotype quality.

^a^ AF ≤0.005, no quality filters.

^b^ Maximum AF observed in public databases for any subpopulation.

**TABLE 5:** Variants in X‐linked genes (recessive inheritance model) meeting filtering criteria.^a^

Case	Gene	Variant	Locus‐reference allele	Allele	GQ	AD	AF^b^	CADD	GDI (%)
10	*IQSEC2*	p.His1246_His1247del	X:53234948‐ATGGTGG	[A]	99	[3, 171]	0	—	34.4
12	*IQSEC2*	p.Pro1109Leu	X:53236447‐G	[A]	76	[2, 40]	0.0002	18.5	34.4
11	*IQSEC2*	p.Gly115Cys	X:53320781‐C	[A]	99	[4, 106]	0.0032	27.5	34.4

*Note*: AD allelic depth for the reference allele (listed first) and the alternate allele (listed second); since three of these infants are male, we expect the depths of the reference allele to be zero for these hemizygous calls; the small number of reference allele depths between 2 and 4 seen here are likely due to sequencing error or other technical artifact.

Abbreviations: AF, allele frequency, CADD, combined annotation dependent depletion score, GDI, gene damage index, GQ, genotype quality.

^a^ AD >10, GQ >50, AF ≤0.005 in public databases, GDI <75th percentile.

^b^ Maximum AF observed in public databases for any subpopulation.

## 4 DISCUSSION

**FIGURE 1:**
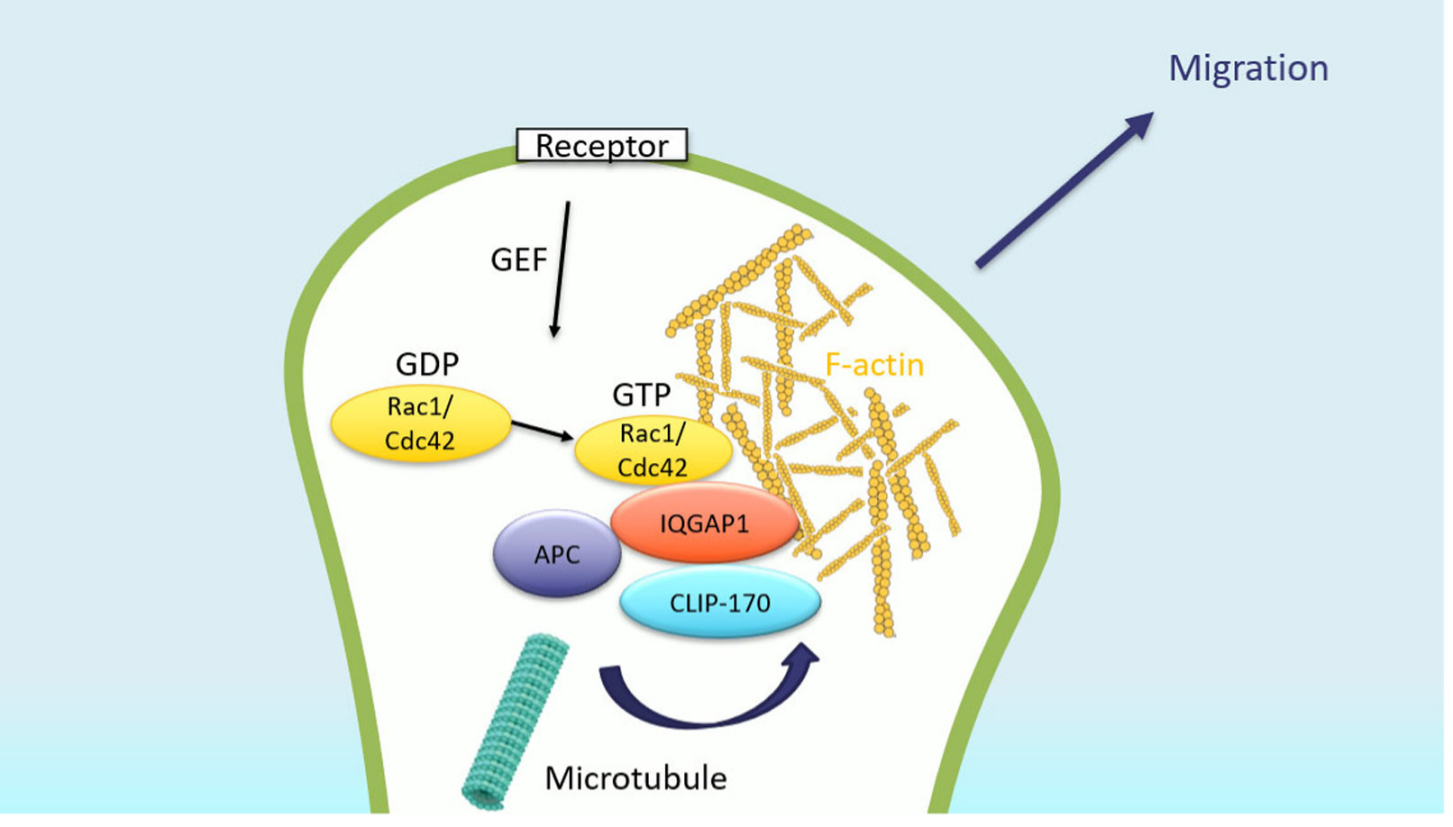
Role of *IQGAP1* in cell polarization and directional cell migration. *APC* adenomatous polyposis coli, *GDP* guanosine diphosphate, *GEF* guanine nucleotide exchange factors, *GTP* guanosine triphosphate. Cell migration is initiated by extracellular signals (GEFs), such as chemokines and growth factors, that activate Rac1 and Cdc42 (GTP‐bound) at the leading edges. Activated Rac1 and Cdc42 induce polymerization of actin filaments and recruit IQGAP1 that crosslinks the actin filaments. Following that, IQGAP1 recruits APC to the actin filaments and IQGAP1, through CLIP‐170, captures the plus‐ends of microtubules. Then, APC stabilizes the microtubules, which are essential for stable actin meshwork at the leading edges.

We identified two genes, *IQGAP1* and *NRCAM*, that have a high number of rare functional variants in the 91 AP cases that were analyzed. Notably, both genes are involved in cell migration. Single heterozygous missense variants in *IQGAP1* were identified in seven cases. IQGAP1 is a scaffold protein participating in multiple cellular functions including actin organization and regulation of cell motility (Bensenor et al., 2007; Figure 1). Cell migration is a multistep process that requires changes to the cytoskeleton (Ridley, 2001). IQGAP1 regulates cytoskeletal function by interacting with Cdc42 and Rac1 (Hart et al., 1996; Joyal et al., 1997), CLIP‐170 (Fukata et al., 2002), and actin (Erickson et al., 1997; Mateer et al., 2002). Cell migration is initiated in response to extracellular signals such as cytokines and growth factors (Choi et al., 2013) that activate Rac1 and Cdc42 (members of the Rho family GTPases); activated Rac1 and Cdc42 recruit IQGAP1, which stabilizes them in the active GTP‐bound state (Brandt et al., 2007; Le Clainche et al., 2007; Swart‐Mataraza et al., 2002). In addition, in directionally migrating cells, IQGAP1 accumulates on the leading edge where it facilitates actin polymerization and directly crosslinks actin filaments (Briggs & Sacks, 2003; Hart et al., 1996; Mataraza et al., 2003). Then, it recruits adenomatous polyposis coli (APC) to actin filaments and captures the plus‐ends of the microtubules through CLIP‐170 (a microtubule‐binding protein; Fukata et al., 2002; Noritake et al., 2005). Following that, APC stabilizes the microtubules, which is essential for the actin meshwork at the leading edge to be stable (Noritake et al., 2005).

*IQGAP1* has also been characterized as an oncogene (Dong et al., 2016; Xia et al., 2014) implicated in several types of cancer including pancreatic (Casteel et al., 2012; Hu et al., 2019; Liu et al., 2010; Wang et al., 2013, 2014; Xia et al., 2014; Zhao et al., 2014). In addition, *IQGAP1* is known to mediate the Wnt/β‐catenin signaling pathway to induce cancer metastasis (Peng et al., 2021). Based on the cancer data, we hypothesize that genetic variants in *IQGAP1* may result in decreased cell migration. Thus, the pancreatic buds may not migrate normally.

Our hypothesis is supported by a report that a knock down of *IQGAP1* by both transient and stable expression of small interfering RNA (siRNA) resulted in significantly slowed cell migration (Mataraza et al., 2003). That study showed that changing the intracellular concentration of *IQGAP1* changed cell migration; overexpression of *IQGAP1* resulted in increased cell motility, while decreased levels decreased cell motility (Mataraza et al., 2003). In addition, in a different study, a dominant negative *IQGAP1* decreased active levels of Cdc42 and Rac1 and so cell motility was decreased significantly (Swart‐Mataraza et al., 2002).

The second gene we identified, *NRCAM*, which was found in seven cases, encodes a neuronal cell adhesion molecule of the superfamily of immunoglobulins (Grumet, 1991, 1997; Grumet et al., 1991; Wang et al., 1998). First described in neuron–neuron adhesion, *NRCAM*, was then reported to potentially play a role in cell–cell communication via signaling from its intracellular domain to the actin cytoskeleton during directional cell migration. Like *IQGAP1*, it is overexpressed in various types of cancer, including pancreatic carcinoma (Dhodapkar et al., 2001; Wang et al., 1998) again suggesting that under‐expression due to genetic variants could interfere with normal migration.

In addition, we provided confirmatory evidence regarding two genes previously reported to be associated with AP: *PDX1* and *FOXF1*. *PDX1* is a key regulatory element of pancreas development (Pethe et al., 2021). The association between AP and the *PDX1* gene was previously suggested in a case study of a patient with AP and other related abnormalities including duodenal atresia, hypoplastic gallbladder, permanent neonatal diabetes mellitus, and exocrine pancreatic insufficiency (Kulkarni et al., 2017). There are reports that mutations in *PDX1* resulted in pancreatic agenesis (Schwitzgebel et al., 2003; Stoffers et al., 1997). In a case report a male patient had AP, while his sibling had a *FOXF1* variant and appeared to have, among other defects, dorsal pancreatic agenesis (Reiter et al., 2016). *FOXF1* has been found to be involved in the development of the foregut (Madison et al., 2009; Mahlapuu et al., 2001).

### 4.1 How do our findings fit to the existing hypotheses?

Going back to the three theories regarding the pathogenesis of AP, it is clear that our findings do not specifically fit any of them. Both genes we identified are involved in actin cytoskeleton which mediates various important cellular processes such as cell migration. This might be a fourth causal pathway that may be relevant in a subset of cases not explained by the previous three theories.

Strengths of our analysis include a well‐characterized, population‐based study sample in which a very high percentage of major malformations were identified. Additionally, our study is the largest exome sequencing study of AP cases to date. Limitations of the study include that only limited amounts of DNA could be recovered from the newborn blood spots. This led to lower coverage for some individuals and also means that all of the DNA available was used for WES, leaving none that could be used for confirmatory Sanger sequencing for variants of interest. That said, we have done a careful review of the raw data using IGV and have included screenshots of them in Supplemental Material. Many technical artifacts leading to spurious genotype calls, such as mismapping, can be observed when visualized in this way, and we did not find any such patterns for the variants reported in our tables. In addition, whether the parents were affected is not known. Lastly, functional studies were not conducted because none of the variants were recurrent. Even though our study has limitations, it presents new data underlying the genetic etiology of non‐syndromic AP to stimulate future research.

## 5 CONCLUSIONS

To our knowledge this is the largest exome sequencing study in children with AP. Fifteen percent of our cases had variants in two genes involved in cell migration. We hypothesize that these variants in *IQGAP1* and *NRCAM* may help explain the failure of the ventral pancreatic bud to migrate. Our results require confirmation, but they may provide new directions for research.

## AUTHOR CONTRIBUTIONS

All the authors have accepted responsibility for the entire content of this submitted manuscript and approved submission. J.L.M, and G.M.S. made the concept. N.P. and J.L. performed and analyzed the sequencing experiments. W.Y. performed the statistical 2 analysis. G.P interpreted the sequencing results and drafted the manuscript. G.P, N.P, J.L, W.Y, S.R, G.M.S, and J.L.M. edited the manuscript.

## ACKNOWLEDGEMENTS

We sincerely thank all the participants who volunteered to take part in this study.

## CONFLICT OF INTEREST STATEMENT

None declared.

## ETHICS STATEMENT

The IRB approvals are already in the manuscript in the methods section.

## FUNDING INFORMATION

This study was funded by the Intramural Research Program, Eunice Kennedy Shriver National Institute of Child Health and Human Development, NIH. NIH/NICHD Task Order HHSN27500011 under Contract HHSN275201300023I.

## Supporting information


Data S1.

Table S1.

## Data Availability

The data are based on DNA derived from newborn bloodspots obtained from the CA BIoBank. Data use agreements do not permit the authors to share individual‐level data with others owing to privacy or ethical restrictions.
