# Supplementary material for: Exome sequencing findings in children with annular pancreas
Source: Mol Genet Genomic Med. 2023 Aug 28;11(10):e2233. doi: 10.1002/mgg3.2233 (PMC10568395; doi:10.1002/mgg3.2233)
Supplement: Supplementary file 2 — Table S1. [file MGG3-11-e2233-s002.docx]

**Supplemental Table 1. Variant filtering criteria^a^**

| Criteria for autosomal dominant variants | - Not in GATK tranche, AD >10, GQ >50 - AF ≤0.005 in public databases - Genes with ≥2 loss-of-function variants or ≥6 loss-of-function or missense variants - GDI <75th percentile |
| --- | --- |
| Criteria for compound heterozygotes | - Not in GATK tranche, AD >10, GQ >50 - AF ≤0.005 in public databases - Genes with ≥2 loss-of-function or missense variants in the same sample that were on separate haplotypes, as determined in IGV. - Genes with ≥2 samples with compound heterozygotes - GDI <75th percentile |
| Criteria for previously reported genes | - ≥1 loss-of-function or missense variants in gene with prior evidence for association with AP - AF ≤0.005 in public databases - No quality filters |
| Criteria for autosomal recessive variants | - Not in GATK tranche, AD >10, GQ >50 - AF ≤0.005 in public databases - Homozygous for loss-of-function or missense variants - GDI <75th percentile |
| Criteria for X-linked recessive variants | - Not in GATK tranche, AD >10, GQ >50 - AF ≤0.005 in public databases - Homozygous (female) or hemizygous (male) loss-of-function or missense variants on chromosome X - GDI <75th percentile |

*AD* allelic depth, *AF* allele frequency, *AP* annular pancreas, *GDI* gene damage index, *GQ* genotype quality, *WGS* whole genome sequencing, *ESP* Exome Sequencing Project

^a^Quality of variants was reviewed in Integrated Genomics Viewer

When filtering by allele frequency (AF), we use the maximum allele frequency from the following populations/public databases, as annotated by ANNOVAR v2018Apr16:October 2014 and August 2015 releases of 1000 genomes - (all + AFR/EUR/EAS), ESP (all + AFR/EUR), 50 thousand internal WGS controls (all + AFR/AMR/EUR/EAS/SAS), ExAC-non-TGCA (all + AFR/AMR/EAS/FIN/NFE/OTHER/SAS), gnomADV2 WGS (all + AFR/AMR/ASJ/EAS/FIN/NFE/OTHER), gnomADV2 WES (all + AFR/AMR/ASJ/EAS/FIN/NFE/OTHER/SAS), gnomADV3 WGS (all + AFR/AMR/ASJ/EAS/FIN/NFE/OTHER/SAS)

**Supplemental Table 2. Additional variants passing the criteria of the autosomal dominant model ordered by gene damage index.**

| Case | Gene | Variant | Locus-Reference Allele | [Allele 1, Allele 2] | GQ | AD | AF^a^ | CADD | GDI |
| --- | --- | --- | --- | --- | --- | --- | --- | --- | --- |
| 14 | *MPV17* | c.461+2T>C | 2:27311897-A | [A, G] | 99 | [30, 25] | 0.0009 | 24.7 | 14.8% |
| 8 | *MPV17* | p.Gly79Arg | 2:27312724-C | [C, G] | 99 | [34, 23] | 0.00018 | 29.9 | 14.8% |
| 13 | *MPV17* | c.227+1G>C | 2:27317077-C | [C, G] | 99 | [72, 63] | 0.0004 | 5.3 | 14.8% |
| 16 | *HIST1H2BM* | p.Met1? | 6:27815044-A | [A, G] | 99 | [48, 38] | 0.0019 | 10.8 | 17.8% |
| 15 | *HIST1H2BM* | p.Met1? | 6:27815046-G | [G, A] | 99 | [37, 40] | 0.0006 | 22.7 | 17.8% |
| 18 | *FAM183A* | p.Glu61Ter | 1:43150808-G | [G, T] | 99 | [27, 18] | 0.0033 | 42.0 | 21.6% |
| 17 | *FAM183A* | p.Trp126Ter | 1:43156285-G | [G, A] | 99 | [30, 18] | 0.0029 | 37.0 | 21.6% |
| 20 | *SUMO4* | p.Met1? | 6:149400393-T | [T, C] | 99 | [25, 26] | 0.0018 | 16.4 | 21.8% |
| 19 | *SUMO4* | p.Ter96TrpextTer23 | 6:149400679-A | [A, G] | 99 | [11, 6] | 0.0015 | 13.7 | 21.8% |
| 21 | *C1orf228* | n.380+1G>A | 1:44689904-G | [G, A] | 99 | [22, 8] | 0.0003 | 26.3 | 27.4% |
| 22 | *C1orf228* | n.380+1G>A | 1:44689904-G | [G, A] | 99 | [12, 10] | 0.0003 | 26.3 | 27.4% |
| 7 | *MAPK13* | p.Arg173Ter | 6:36136677-C | [C, T] | 99 | [71, 69] | 0.0019 | 36.0 | 30.8% |
| 1 | *MAPK13* | p.Gln216Ter | 6:36138737-C | [C, T] | 99 | [66, 55] | 0.0011 | 13.0 | 30.8% |
| 23 | *MAPK13* | p.Ala282Val | 6:36138882-C | [C, T] | 99 | [62, 52] | 0.0043 | 0.0 | 30.8% |
| 24 | *LDHAL6A* | c.592+1G>A | 11:18475640-G | [G, A] | 99 | [52, 40] | 0.0041 | 23.9 | 36.4% |
| 25 | *LDHAL6A* | c.592+1G>A | 11:18475640-G | [G, A] | 99 | [9, 10] | 0.0041 | 23.9 | 36.4% |
| 27 | *ANKFY1* | p.Tyr1125His | 17:4170754-A | [A, G] | 99 | [43, 40] | 0.0041 | 25.2 | 40.1% |
| 28 | *ANKFY1* | p.Ala1104Thr | 17:4170817-C | [C, T] | 99 | [9, 9] | 0.0003 | 27.5 | 40.1% |
| 26 | *ANKFY1* | p.Asp663Asn | 17:4183489-C | [C, T] | 95 | [7, 4] | 0.0015 | 16.6 | 40.1% |
| 24 | *ANKFY1* | p.Glu422Ter | 17:4195437-C | [C, A] | 99 | [32, 53] | 0.00009 | 37.0 | 40.1% |
| 29 | *ANKFY1* | c.136+1G>A | 17:4263594-C | [C, T] | 86 | [10, 6] | 0.0003 | 24.7 | 40.1% |
| 36 | *MMP19* | p.Arg408Ter | 12:55837341-G | [G, A] | 99 | [25, 37] | 0.0039 | 34.0 | 47.3% |
| 37 | *MMP19* | c.173+1G>A | 12:55842352-C | [C, T] | 99 | [53, 43] | 0.0023 | 29.2 | 47.3% |
| 39 | *NUDT16* | p.Gly3Ter | 3:131381811-G | [G, T] | 99 | [69, 54] | 0.0042 | 29.4 | 47.3% |
| 3 | *NUDT16* | p.Leu27Phe | 3:131381883-C | [C, T] | 99 | [67, 48] | 0.00001 | 23.7 | 47.3% |
| 18 | *NUDT16* | p.His99GlnfsTer17 | 3:131382203-AC | [AC, A] | 99 | [84, 59] | 0.0046 | . | 47.3% |
| 38 | *NUDT16* | p.Pro133Leu | 3:131383360-C | [C, T] | 99 | [23, 44] | 0 | 16.8 | 47.3% |
| 25 | *BAG1* | p.Ter346 | 9:33255219-TC | [TC, T] | 81 | [23, 4] | 0 | . | 49.1% |
| 40 | *BAG1* | p.Gln120Ter | 9:33264317-G | [G, A] | 99 | [52, 68] | 0.0001 | 24.2 | 49.1% |
| 31 | *BAG1* | p.Ser33Trp | 9:33264577-G | [G, C] | 99 | [34, 30] | 0.0042 | 25.8 | 49.1% |
| 42 | *KIF13B* | p.Gly1683Trp | 8:29071791-C | [C, A] | 99 | [67, 46] | 0.00009 | 24.9 | 60.2% |
| 41 | *KIF13B* | p.Ser1376Arg | 8:29109467-G | [G, C] | 99 | [17, 18] | 0.0012 | 3.4 | 60.2% |
| 40 | *KIF13B* | p.Gly827Glu | 8:29140472-C | [C, T] | 99 | [35, 23] | 0 | 32.0 | 60.2% |
| 44 | *KIF13B* | p.Asn738Ser | 8:29146352-T | [T, C] | 99 | [11, 21] | 0.001 | 4.2 | 60.2% |
| 43 | *KIF13B* | p.Lys394Gln | 8:29165751-T | [T, G] | 99 | [14, 27] | 0.0016 | 21.7 | 60.2% |
| 3 | *KIF13B* | p.Asp338Gly | 8:29167518-T | [T, C] | 99 | [21, 24] | 0.00009 | 27.0 | 60.2% |
| 37 | *KIF13B* | p.Lys165Asn | 8:29186294-T | [T, A] | 99 | [53, 40] | 0 | 24.1 | 60.2% |
| 41 | *TSHZ2* | c.31+1G>C | 20:53185677-G | [G, C] | 51 | [3, 10] | 0.0047 | 0.4 | 60.4% |
| 22 | *TSHZ2* | p.Glu34Gly | 20:53253559-A | [A, G] | 99 | [7, 9] | 0.0008 | 23.3 | 60.4% |
| 48 | *TSHZ2* | p.Ala425Thr | 20:53254731-G | [G, A] | 99 | [7, 8] | 0.00009 | 21.6 | 60.4% |
| 45 | *TSHZ2* | p.Val479Met | 20:53254893-G | [G, A] | 99 | [30, 33] | 0.0036 | 0.3 | 60.4% |
| 46 | *TSHZ2* | p.Met590Thr | 20:53255227-T | [T, C] | 99 | [73, 85] | 0 | 0.1 | 60.4% |
| 14 | *TSHZ2* | p.Ala610Val | 20:53255287-C | [C, T] | 99 | [57, 63] | 0.001 | 0.0 | 60.4% |
| 36 | *TSHZ2* | p.Pro818Arg | 20:53255911-C | [C, G] | 99 | [53, 47] | 0.0003 | 20.6 | 60.4% |
| 47 | *TSHZ2* | p.Ser836Pro | 20:53255964-T | [T, C] | 99 | [37, 35] | 0.0005 | 25.2 | 60.4% |
| 50 | *GPNMB* | p.His178ProfsTer15 | 7:23257056-CA | [CA, C] | 99 | [23, 22] | 0.0002 | . | 63.4% |
| 51 | *GPNMB* | p.Arg189Ter | 7:23260003-C | [C, T] | 99 | [32, 33] | 0.003 | 36.0 | 63.4% |
| 49 | *GPNMB* | p.Tyr281Cys | 7:23260597-A | [A, G] | 99 | [33, 22] | 0.0004 | 26.4 | 63.4% |
| 52 | *GPNMB* | p.Cys323Ser | 7:23260723-G | [G, C] | 99 | [35, 23] | 0.0018 | 24.7 | 63.4% |
| 54 | *SRCAP* | p.Val1541Met | 16:30724045-G | [G, A] | 99 | [16, 11] | 0.0041 | 10.0 | 63.4% |
| 13 | *SRCAP* | p.Ser1553Leu | 16:30724082-C | [C, T] | 99 | [50, 39] | 0.0002 | 19.2 | 63.4% |
| 14 | *SRCAP* | p.Val2222Met | 16:30734550-G | [G, A] | 99 | [15, 14] | 0.0039 | 22.7 | 63.4% |
| 53 | *SRCAP* | p.Pro2507Ser | 16:30737559-C | [C, T] | 99 | [56, 52] | 0.0009 | 12.7 | 63.4% |
| 33 | *SRCAP* | p.Met2796Thr | 16:30738427-T | [T, C] | 99 | [78, 75] | 0.0033 | 0.1 | 63.4% |
| 57 | *SRCAP* | p.Gly2839Ser | 16:30738555-G | [G, A] | 99 | [6, 4] | 0.0023 | 7.4 | 63.4% |
| 55 | *SRCAP* | p.Val2895Phe | 16:30738723-G | [G, T] | 99 | [120, 71] | 0.00002 | 13.7 | 63.4% |
| 56 | *SRCAP* | p.Leu3163Val | 16:30739527-C | [C, G] | 99 | [31, 19] | 0.0015 | 8.7 | 63.4% |
| 44 | *NUP188* | p.Gly6Ala | 9:128947736-G | [G, C] | 59 | [3, 7] | 0.0041 | 6.5 | 63.8% |
| 24 | *NUP188* | p.Ala377Val | 9:128973176-C | [C, T] | 99 | [54, 36] | 0.0004 | 24.6 | 63.8% |
| 17 | *NUP188* | p.Thr498Lys | 9:128981367-C | [C, A] | 99 | [12, 12] | 0.00002 | 23.4 | 63.8% |
| 58 | *NUP188* | p.Asp943His | 9:128993383-G | [G, C] | 99 | [10, 9] | 0.0012 | 28.3 | 63.8% |
| 59 | *NUP188* | c.4510-1G>T | 9:129005302-G | [G, T] | 99 | [46, 40] | 0.00033 | 24.7 | 63.8% |
| 13 | *NUP188* | p.Thr1644Ile | 9:129006111-C | [C, T] | 99 | [51, 30] | 0.0009 | 18.5 | 63.8% |
| 15 | *NUP188* | c.5073+1G>T | 9:129006369-G | [G, T] | 99 | [105, 86] | 0 | 26.8 | 63.8% |
| 33 | *SPATA22* | c.173-2A>G | 17:3462769-T | [T, C] | 99 | [8, 5] | 0.0005 | 24.1 | 65.9% |
| 60 | *SPATA22* | c.173-2A>G | 17:3462769-T | [T, C] | 99 | [10, 8] | 0.0005 | 24.1 | 65.9% |
| 27 | *LRP1* | p.Ser687Thr | 12:57160972-T | [T, A] | 99 | [67, 53] | 0 | 18.1 | 66.0% |
| 61 | *LRP1* | p.Pro938Ser | 12:57166944-C | [C, T] | 99 | [22, 50] | 0.001 | 21.6 | 66.0% |
| 13 | *LRP1* | p.Arg1625His | 12:57179464-G | [G, A] | 99 | [131, 97] | 0.0035 | 22.7 | 66.0% |
| 12 | *LRP1* | p.Arg2387His | 12:57190933-G | [G, A] | 99 | [63, 60] | 0.0001 | 33.0 | 66.0% |
| 24 | *LRP1* | p.Gly3725Glu | 12:57204729-G | [G, A] | 99 | [64, 65] | 0.0029 | 23.3 | 66.0% |
| 17 | *LRP1* | p.Arg3942Gln | 12:57206707-G | [G, A] | 99 | [38, 26] | 0.0001 | 24.2 | 66.0% |
| 44 | *LRP1* | p.Ser3977Leu | 12:57208108-C | [C, T] | 99 | [115, 82] | 0.0016 | 29.8 | 66.0% |
| 62 | *NXPE2* | c.26+2T>G | 11:114678603-T | [T, G] | 99 | [11, 7] | 0.0002 | 12.1 | 66.3% |
| 52 | *NXPE2* | p.Trp529Ter | 11:114706837-G | [G, A] | 99 | [35, 36] | 0.0017 | 39.0 | 66.3% |
| 63 | *MICAL2* | p.Arg386Gln | 11:12220409-G | [G, A] | 99 | [57, 48] | 0.0003 | 29.3 | 68.0% |
| 39 | *MICAL2* | p.Arg1014Gln | 11:12256870-G | [G, A] | 99 | [86, 75] | 0.0043 | 34.0 | 68.0% |
| 5 | *MICAL2* | p.Ala1040Thr | 11:12256947-G | [G, A] | 99 | [72, 56] | 0.0035 | 11.1 | 68.0% |
| 7 | *MICAL2* | p.Ala1040Thr | 11:12256947-G | [G, A] | 99 | [88, 79] | 0.0035 | 11.1 | 68.0% |
| 46 | *MICAL2* | p.Ala1040Thr | 11:12256947-G | [G, A] | 99 | [73, 86] | 0.0035 | 11.1 | 68.0% |
| 27 | *MICAL2* | p.Gly897Asp | 11:12259931-G | [G, A] | 99 | [72, 53] | 0.002 | 19.1 | 68.0% |
| 41 | *MICAL2* | p.Lys974Arg | 11:12260162-A | [A, G] | 99 | [48, 44] | 0.0013 | 12.6 | 68.0% |
| 45 | *SLC15A1* | p.Phe605Ile | 13:98687595-A | [A, T] | 83 | [6, 16] | 0.0025 | 29.0 | 68.0% |
| 33 | *SLC15A1* | p.Phe427LeufsTer2 | 13:98704423-TA | [TA, T] | 99 | [29, 30] | 0.000059 | . | 68.0% |
| 47 | *SLC15A1* | c.1068-1G>C | 13:98708768-C | [C, G] | 99 | [14, 20] | 0.0001 | 23.9 | 68.0% |
| 66 | *ARID1B* | p.Lys26del | 6:156778004-CAAG | [CAAG, C] | 99 | [15, 9] | 0.0001 | . | 68.6% |
| 47 | *ARID1B* | p.Ala336Thr | 6:156778935-G | [G, A] | 99 | [40, 26] | 0.0003 | 11.4 | 68.6% |
| 18 | *ARID1B* | p.Met973Thr | 6:157167078-T | [T, C] | 99 | [27, 30] | 0.0029 | 23.0 | 68.6% |
| 64 | *ARID1B* | p.Gln687Glu | 6:157186480-C | [C, G] | 99 | [24, 33] | 0.0017 | 1.7 | 68.6% |
| 60 | *ARID1B* | p.Gln687His | 6:157186482-G | [G, T] | 60 | [9, 4] | 0.000082 | 1.6 | 68.6% |
| 21 | *ARID1B* | p.Gln1437Glu | 6:157200903-C | [C, G] | 99 | [50, 32] | 0.00003 | 6.3 | 68.6% |
| 56 | *ARID1B* | p.Ser1455Pro | 6:157200957-T | [T, C] | 99 | [27, 12] | 0.0001 | 17.3 | 68.6% |
| 25 | *ARID1B* | p.Pro1849Arg | 6:157206687-C | [C, G] | 99 | [9, 10] | 0.0012 | 16.0 | 68.6% |
| 65 | *ARID1B* | p.Asn1915His | 6:157206884-A | [A, C] | 99 | [45, 34] | 0.000092 | 23.1 | 68.6% |
| 23 | *PIWIL3* | p.Arg873His | 22:24719503-C | [C, T] | 99 | [16, 18] | 0.0004 | 6.5 | 69.3% |
| 57 | *PIWIL3* | p.Arg185Trp | 22:24756508-G | [G, A] | 99 | [11, 14] | 0.003 | 24.3 | 69.3% |
| 45 | *PIWIL3* | p.Gln36ArgfsTer41 | 22:24759985-TG | [TG, T] | 99 | [17, 9] | 0.0041 | . | 69.3% |
| 67 | *PIWIL3* | p.Gln36ArgfsTer41 | 22:24759985-TG | [TG, T] | 99 | [7, 5] | 0.0041 | . | 69.3% |
| 63 | *ALS2CR11* | p.Glu1620TyrfsTer6 | 2:201491479-ATTTC | [ATTTC, A] | 99 | [10, 4] | 0.0034 | . | 69.3% |
| 68 | *ALS2CR11* | p.Gln730ThrfsTer24 | 2:201494153-G | [G, GT] | 99 | [13, 6] | 0.0002 | . | 69.3% |
| 50 | *COBL* | p.Met896Ile | 7:51028579-C | [C, T] | 99 | [94, 86] | 0.0018 | 0.0 | 71.5% |
| 69 | *COBL* | p.Ile821Thr | 7:51028805-A | [A, G] | 99 | [45, 26] | 0.0002 | 12.9 | 71.5% |
| 6 | *COBL* | p.Gly803Ser | 7:51028860-C | [C, T] | 99 | [50, 27] | 0.0018 | 7.6 | 71.5% |
| 6 | *COBL* | p.Ala801Thr | 7:51028866-C | [C, T] | 99 | [26, 38] | 0.0002 | 0.2 | 71.5% |
| 63 | *COBL* | p.His590Tyr | 7:51029499-G | [G, A] | 99 | [29, 29] | 0.0007 | 0.2 | 71.5% |
| 12 | *COBL* | p.Gly411Asp | 7:51083093-C | [C, T] | 99 | [15, 8] | 0.0006 | 13.5 | 71.5% |
| 51 | *COBL* | p.Pro148Leu | 7:51193392-G | [G, A] | 99 | [12, 10] | 0.0019 | 8.6 | 71.5% |
| 38 | *COBL* | p.His41Tyr | 7:51219865-G | [G, A] | 99 | [36, 32] | 0.00011 | 10.6 | 71.5% |
| 41 | *NT5DC4* | p.Tyr61Ter | 2:112722083-C | [C, A] | 99 | [98, 131] | 0 | 25.5 | 71.9% |
| 40 | *NT5DC4* | p.Ala101Thr | 2:112722499-G | [G, A] | 99 | [48, 24] | 0.0007 | 13.2 | 71.9% |
| 70 | *NT5DC4* | p.Ala101Thr | 2:112722499-G | [G, A] | 99 | [44, 49] | 0.0007 | 13.2 | 71.9% |
| 71 | *NT5DC4* | p.Trp104Ter | 2:112722509-G | [G, A] | 54 | [4, 11] | 0.0001 | 36.0 | 71.9% |
| 43 | *DLG5* | p.Arg890His | 10:77821815-C | [C, T] | 99 | [18, 13] | 0.0024 | 21.6 | 71.9% |
| 41 | *DLG5* | p.Ile811Thr | 10:77822052-A | [A, G] | 99 | [41, 30] | 0.0003 | 21.4 | 71.9% |
| 44 | *DLG5* | p.Asn546Ile | 10:77834025-T | [T, A] | 58 | [10, 4] | 0.003 | 25.4 | 71.9% |
| 57 | *DLG5* | p.Asn546Ile | 10:77834025-T | [T, A] | 51 | [12, 4] | 0.003 | 25.4 | 71.9% |
| 16 | *DLG5* | p.Thr387Met | 10:77842158-G | [G, A] | 99 | [36, 34] | 0.002 | 24.7 | 71.9% |
| 39 | *DLG5* | p.Thr387Met | 10:77842158-G | [G, A] | 99 | [24, 33] | 0.002 | 24.7 | 71.9% |
| 25 | *DLG5* | p.Ile85Val | 10:77926268-T | [T, C] | 99 | [9, 7] | 0.003 | 7.8 | 71.9% |
| 53 | *DLG5* | p.Ile85Val | 10:77926268-T | [T, C] | 99 | [92, 53] | 0.003 | 7.8 | 71.9% |
| 18 | *PCK2* | p.Tyr26Ter | 14:24094814-C | [C, G] | 99 | [42, 27] | 0 | . | 72.6% |
| 72 | *PCK2* | p.Glu43Asp | 14:24095162-A | [A, C] | 99 | [93, 58] | 0.0004 | . | 72.6% |
| 8 | *PCK2* | p.Arg193Ter | 14:24098591-C | [C, T] | 99 | [31, 24] | 0.0009 | 39.0 | 72.6% |
| 63 | *PCK2* | p.Ile237AspfsTer40 | 14:24099091-T | [T, TA] | 99 | [54, 54] | 0.0008 | . | 72.6% |
| 59 | *PCK2* | p.Arg560Gln | 14:24103720-G | [G, A] | 99 | [45, 32] | 0.0029 | 29.2 | 72.6% |
| 30 | *NUP98* | p.Arg1739Cys | 11:3676347-G | [G, A] | 99 | [81, 59] | 0.0006 | 34.0 | 72.8% |
| 75 | *NUP98* | p.Thr1521Ile | 11:3686087-G | [G, A] | 99 | [6, 14] | 0 | 23.4 | 72.8% |
| 22 | *NUP98* | p.Asp1214Asn | 11:3700712-C | [C, T] | 99 | [31, 30] | 0.0032 | 25.9 | 72.8% |
| 74 | *NUP98* | p.Ala1137Ser | 11:3702566-C | [C, A] | 99 | [22, 43] | 0.0009 | 25.1 | 72.8% |
| 45 | *NUP98* | p.Arg1025His | 11:3705208-C | [C, T] | 99 | [6, 4] | 0.0029 | 23.4 | 72.8% |
| 76 | *NUP98* | p.Thr899Met | 11:3712610-G | [G, A] | 99 | [24, 19] | 0.0043 | 19.4 | 72.8% |
| 73 | *NUP98* | p.Asn665Ser | 11:3723309-T | [T, C] | 99 | [24, 25] | 0.0005 | 0.0 | 72.8% |
| 3 | *URB1* | p.Pro2232Leu | 21:32315039-G | [G, A] | 99 | [25, 20] | 0.0003 | 16.9 | 73.0% |
| 7 | *URB1* | p.Arg1716Trp | 21:32324578-G | [G, A] | 99 | [13, 13] | 0.0004 | 35.0 | 73.0% |
| 13 | *URB1* | p.Arg1286Cys | 21:32346968-G | [G, A] | 99 | [65, 54] | 0.0008 | 23.3 | 73.0% |
| 30 | *URB1* | p.Val572Met | 21:32361049-C | [C, T] | 99 | [24, 11] | 0.0036 | 9.2 | 73.0% |
| 60 | *URB1* | p.Pro537Leu | 21:32361921-G | [G, A] | 99 | [13, 12] | 0.0018 | 8.4 | 73.0% |
| 50 | *URB1* | p.Leu505Ser | 21:32362017-A | [A, G] | 99 | [68, 57] | 0.0017 | 29.5 | 73.0% |
| 67 | *URB1* | p.Ala410Gly | 21:32366724-G | [G, C] | 99 | [5, 5] | 0.0003 | 26.7 | 73.0% |
| 77 | *URB1* | p.Trp390Ter | 21:32368430-C | [C, T] | 99 | [26, 33] | 0.00025 | 39.0 | 73.0% |
| 28 | *AGBL1* | p.Met1? | 15:86079975-G | [G, A] | 93 | [4, 10] | 0 | . | 73.4% |
| 6 | *AGBL1* | p.Glu23Ter | 15:86142019-G | [G, T] | 99 | [38, 45] | 0.0041 | . | 73.4% |
| 52 | *AGBL1* | p.Ser523Cys | 15:86264739-C | [C, G] | 99 | [26, 28] | 0.0031 | 26.4 | 73.4% |
| 43 | *AGBL1* | p.Asn589Ser | 15:86267004-A | [A, G] | 99 | [25, 20] | 0.0003 | 0.0 | 73.4% |
| 78 | *AGBL1* | p.Asp623GlufsTer2 | 15:86269948-AT | [AT, A] | 99 | [10, 12] | 0.00033 | . | 73.4% |
| 14 | *AGBL1* | p.Gly851Ser | 15:86397542-G | [G, A] | 99 | [10, 18] | 0.0001 | 34.0 | 73.4% |
| 37 | *AGBL1* | p.Ser870Asn | 15:86522863-G | [G, A] | 99 | [26, 23] | 0.0026 | 0.0 | 73.4% |
| 14 | *ABCC3* | p.Ala41Val | 17:50635291-C | [C, T] | 99 | [29, 40] | 0.0006 | 9.2 | 73.8% |
| 73 | *ABCC3* | p.Ala41Val | 17:50635291-C | [C, T] | 99 | [17, 38] | 0.0006 | 9.2 | 73.8% |
| 22 | *ABCC3* | p.Tyr370Cys | 17:50663791-A | [A, G] | 99 | [31, 23] | 0.0012 | 17.7 | 73.8% |
| 78 | *ABCC3* | p.Gln436Lys | 17:50664079-C | [C, A] | 99 | [20, 18] | 0.0023 | 26.6 | 73.8% |
| 57 | *ABCC3* | p.Ser486Leu | 17:50667579-C | [C, T] | 99 | [24, 23] | 0.0004 | 23.3 | 73.8% |
| 72 | *ABCC3* | p.Pro1222Leu | 17:50678030-C | [C, T] | 99 | [46, 41] | 0.0041 | . | 73.8% |
| 74 | *ABCC3* | p.Ala1481Pro | 17:50687696-G | [G, C] | 99 | [91, 55] | 0 | 25.5 | 73.8% |

*AD* allelic depth for the [Allele1, Allele2], *AF* allele frequency, *CADD* combined annotation dependent depletion score, *GDI* gene damage index, *GQ* genotype quality

^a^Maximum allele frequency observed in any public database for any subpopulation.
